# Influenza vaccine effect on risk of stroke occurrence: a systematic review and meta-analysis

**DOI:** 10.3389/fneur.2023.1324677

**Published:** 2024-01-10

**Authors:** Jalal A. Zahhar, Hassan K. Salamatullah, Maher B. Almutairi, Dania E. Faidah, Lena M. Afif, Toka A. Banjar, Nayef Alansari, Manar Betar, Saeed Alghamdi, Seraj Makkawi

**Affiliations:** ^1^College of Medicine, King Saud Bin Abdulaziz University for Health Sciences, Jeddah, Saudi Arabia; ^2^King Abdullah International Medical Research Center, Jeddah, Saudi Arabia; ^3^Neuroscience Department, King Faisal Specialist Hospital and Research Center, Jeddah, Saudi Arabia; ^4^Department of Neurosciences, Ministry of the National Guard-Health Affairs, Jeddah, Saudi Arabia

**Keywords:** ischemic stroke, hemorrhagic stroke, acute cerebrovascular accident, influenza vaccine, stroke occurrence

## Abstract

**Background:**

Stroke is a significant global cause of mortality and long-term disability, potentially influenced by infections that heighten systemic inflammation and thrombotic events. The full impact of influenza vaccination on stroke remains uncertain. This systematic review and meta-analysis aimed to investigate the association between influenza immunization and stroke incidence.

**Methods:**

We searched for randomized controlled trials (RCTs), case–control, and cohort studies published in PubMed/Medline, Cochrane-Central-Register-of-Controlled-Trials (CENTRAL), and Embase until 5 December 2022, and identified articles investigating the effect of influenza vaccine on stroke occurrence. All articles were screened by two independent reviewers. We performed a meta-analysis to investigate the risk of stroke occurrence in vaccinated vs. unvaccinated individuals. The random-effects model was used in all statistical analyses.

**Results:**

Among the 26 articles meeting our criteria, 10 were retrospective cohort studies, 9 were case–control studies, 3 were prospective cohort studies, 3 were RCTs and 1 case-series. Overall, the studies showed a significant decrease in the risk of stroke incidence/hospitalization among vaccinated patients (OR = 0.81, 95% CI [0.77–0.86], *p* = 0.00001). Furthermore, studies showed flu vaccine decreases the occurrence of mortality among stroke patients (OR = 0.50, 95% CI [0.37–0.68], *p* = 0.00001). Sub-group analysis revealed significant protective effect for patients with specific comorbidities including atrial fibrillation (OR = 0.68, 95% CI [0.57–0.81], *p* = 0.0001), diabetes (OR = 0.76, 95% CI [0.66–0.87], *p* = 0.0001), Chronic obstructive pulmonary disease (OR = 0.70, 95% CI [0.61–0.81], *p* = 0.00001), and hypertension (OR = 0.76, 95% CI [0.70–83], *p* = 0.00001).

**Conclusion:**

The current meta-analysis further supports prior findings that influenza vaccination reduces stroke risk, particularly in patients with comorbidities. Guidelines should promote vaccination for at-risk individuals.

## 1 Introduction

Stroke, a neurological condition caused by an interruption in cerebral blood perfusion, can be broadly classified as either hemorrhagic or ischemic (1). In 2019, stroke was one of leading causes of death worldwide (2). In addition to the significant mortality associated with stroke, the substantial morbidity also leaves up to 50% of survivors with long term disabilities (3). In the Kingdom of Saudi Arabia (KSA), the pooled annual incidence of stroke is equivalent to 29 cases per 100,000 people (4). Therefore, it is crucial to identify the risk factors leading to stroke cases to establish preventive measures. Apart from the conventional risk factors for stroke, including hypertension, diabetes, high blood cholesterol, smoking, and aging (5), there are other identified risk factors, such as infections, which pose both a chronic threat and an acute trigger for stroke (6). The incidence of stroke and other cardiovascular diseases (CVD) is more frequent in winter and during influenza epidemics (7). Moreover, research shows that stroke patients have a higher rate of preceding respiratory infections (8). Studies have also shown that systemic inflammation and infections have a major role in clot formation, and are known to increase circulating inflammatory markers, such as C-reactive protein, and could predict the risk of thrombotic events in humans (9). To test this hypothesis, a study in the United Kingdom (UK) looked at the incidence of myocardial infarction (MI) and stroke after influenza and other vaccinations or after naturally occurring infections, using the United Kingdom General Practice Research Database (GPRD) and the self-controlled case-series method (10). They found there was no increase in the risk of myocardial infarction or stroke in the period following influenza, tetanus, or pneumococcal vaccination. However, the risks of both events were higher after a diagnosis of systemic respiratory tract infection and were highest during the first 3 days. Furthermore, a meta-analysis by Barnes et al. indicated that influenza infection was significantly associated with CVD. Therefore, immunization against influenza can possibly reduce stroke and other vascular events (11). Although the association between influenza vaccines and stroke has been proposed in multiple studies, the results are inconsistent. Some studies found a possible reduction in risk (12–16). Conversely, the protective effects of the vaccine against cerebrovascular diseases have not been established in others (17, 18). Therefore, there is no definitive conclusion regarding the relationship between stroke risk and influenza vaccination. This systematic review and meta-analysis aimed to evaluate the association between receiving the influenza vaccine and reducing stroke incidence.

## 2 Methods

This study followed the Preferred Reporting Items for Systematic Reviews and Meta-Analysis (PRISMA) standard and was registered in PROSPERO before a preliminary search (CRD42022377208).

### 2.1 Search strategy

A complete and comprehensive search of articles published in PubMed/Medline, Cochrane Central Register of Controlled Trials (CENTRAL), and Embase was performed until 5 December 2022. Mesh terms that were used for the search in the databases included (“stroke” OR “cerebrovascular disease” OR “cerebrovascular accident” OR “Ischemic attack” OR “infarction posterior cerebral artery” OR “infarction anterior cerebral artery” OR “Brain Ischemia”) AND (“flu vaccine” OR “influenza vaccine” OR “flu vaccination” OR “influenza vaccination”). We also reviewed the reference lists of relevant articles to ensure that any papers not captured in the database searches were covered.

### 2.2 Criteria for inclusion and exclusion of studies

Studies were considered if they utilized specific designs, such as randomized controlled trials (RCTs), case–control, and cohort studies. Studies that have been performed on patients aged 18 or over who received the influenza vaccine were included; pneumococcal vaccine and any other vaccine other than the influenza vaccine were excluded. Additionally, articles that reported the risk of stroke after receiving the influenza vaccine and measured it by hazard ratio, risk ratio, or odds ratio with the corresponding confidence interval were included. The English language was the only language considered.

### 2.3 Data extraction and study selection

All the included articles were independently evaluated by two investigators and conflicts were resolved by a consensus or a third author consultation. The data that were extracted are the following: first author name, study design, year of conducting the study, country where the study done, inclusion and exclusion criteria of the study, population number, sample size of each arm (influenza vaccine / unvaccinated), range of age included in the study, mean age in the influenza vaccine arm, mean age of the unvaccinated arm, gender (of the whole population), gender of the influenza vaccine arm, gender of the unvaccinated arm, duration of follow up in months, previous stroke in the influenza vaccine arm, previous stroke in the unvaccinated arm, route of vaccine administration, type of event (first stroke, recurrent stroke, both or unspecified), type of stroke, adjusted effect measure of the association between the vaccine and stroke with its confidence interval, the adjusted variables for the adjusted effect measure, concurrent comorbidities [atrial fibrillation, Chronic obstructive pulmonary disease (COPD), diabetes mellitus (DM), hypertension, and smoking].

### 2.4 Risk of bias and quality assessment

Two independent reviewers used the Newcastle-Ottawa Scale (NOS) to assess the methodological quality of the included observational studies, conflicts were resolved by a consensus or a third author consultation. Studies that scored 7–9 are high quality studies with a low risk of bias, a score of 4–6 indicates a fair quality study with a moderate risk of bias, and a score of 0–3 indicates a low-quality study with a high risk of bias. The revised Cochrane risk of bias tool was utilized to assess the risk of bias of the included RCTs. Conflict was resolved with a third author consultation.

### 2.5 Statistical analysis

RevMan (Review Manager) version 5.4.1 (Cochrane Collaboration) was used to perform the data analysis. The random-effects model was used in all statistical analyses. For statistical significance, *p* < 0.05 was set as the upper limit with a 95% confidence level. The I^2^ and *p*-values from the chi-square test were used to evaluate the statistical heterogeneity. The reported effect size in the included studies that represent the incidence/hospitalization due to stroke and mortality were collected. The generic inverse variance method was performed to pool the effect sizes collected from the included studies, and the odds ratio (OR) was used to represent the pooled results. In case the heterogeneity was >50% for the incidence/hospitalization due to stroke and mortality outcomes, sensitivity analysis was conducted to test the robustness of the results. Subgroup analysis was undertaken based on study location, study design, prevention type, and type of stroke. A funnel plot was assessed visually for publication bias.

## 3 Results

### 3.1 Flow chart results

**Figure 1:**
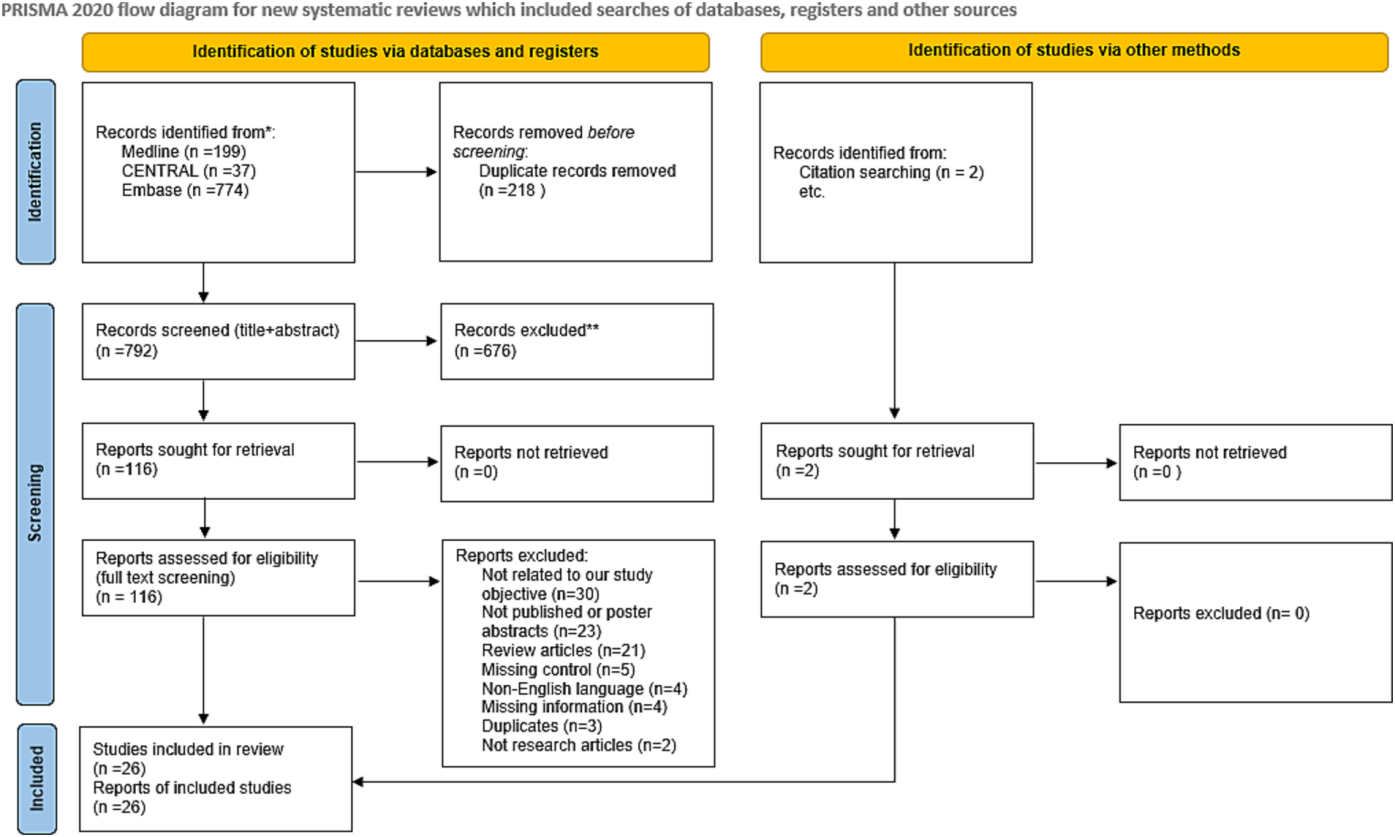
Flow chart of study selection.

The initial systematic literature search of the databases retrieved 1,010 articles. After excluding 218 duplicates and studies with overlapping data using EndNote, 792 studies remained. Irrelevant studies were eliminated by screening the titles and abstracts. The full text of the remaining 116 studies was inspected carefully, and 92 articles were eliminated because they did not fulfill the eligibility criteria. Twenty-four articles were included in the meta-analysis, with an additional two articles identified from the citation screening of the included articles. A total of 26 articles were included in the meta-analysis. Figure 1 summarizes the screening and selection process.

### 3.2 Basic characteristics

Table 1 summarizes the baseline characteristics of the included studies. In total, 6,196,668 patients met the inclusion criteria. With regard to study design, 10 studies were retrospective cohort studies (20, 25–27, 29–32, 34, 35), nine were case–control studies (10, 12, 15–17, 19, 21, 22, 28, 36), three were prospective cohort studies (14, 18, 23), three were randomized clinical trials (13, 24, 33), and one was a case-series (10). Eleven studies included patients aged 65 years and older (12, 14, 19, 20, 22, 23, 28–32), and 13 studies included patients aged < 65 years (10, 13, 15, 16, 18, 24–27, 33–36). 18 studies investigated both ischemic and hemorrhagic stroke, 7 focused on ischemic stroke, and 1 focused only on hemorrhagic stroke.

**Table 1:** Baseline characteristics of included studies.

Author Name. Year	Country	Study design	No. of vaccinated and unvaccinated	Setting/type of patients	participant age	Type of stroke
Ohmit et al. 1995 (19)	USA	Case–control	Cases: 771 vaccinated/562 unvaccinated	Inpatient	≥65 years old	Any stroke
			Controls: 1,664 vaccinated/1,314 unvaccinated			
Lavallee et al. 2002 (16)	France	Case–control	Cases: 42 vaccinated/48 unvaccinated	Inpatient	≥60 years old	Ischemic stroke
			Controls: 107 vaccinated/73 unvaccinated			
Nichol et al. 2003 (20)	USA	Retrospective	165,095 vaccinated/121,288 unvaccinated	Elderly patients	≥65 years old	Any stroke
Smeeth et al. 2004 (10)	UK	Self-control case-series	19,063 vaccinated	Population based	≥18 years old	Any stroke
Grau et al. 2005 (21)	Germany	Case–control	Cases: 71 vaccinated/299 unvaccinated	Inpatient	NR	Hemorrhagic and Ischemic stroke
			Controls: 116 vaccinated/254 unvaccinated			
Wang et al. 2007 (14)	Taiwan	Prospective cohort	35,637 vaccinated/67,061 unvaccinated	Elderly patients	≥65 years old	Any stroke
Puig-Barberà et al. 2007 (22)	Spain	Case–control	Cases: 355 vaccinated/121 unvaccinated	Inpatient	≥65 years old	Any stroke
			Controls: 616 vaccinated/209 unvaccinated			
Piñol-Ripoll et al. 2008 (17)	Spain	Case–control	Cases: 211 vaccinated/182 unvaccinated	Inpatient	NR	Ischemic stroke
			Controls: 220 vaccinated/173 unvaccinated			
Hung et al. 2010 (23)	Hong Kong, China	Prospective cohort	2,076 vaccinated/25,393 unvaccinated	Outpatient	≥65 years old	Ischemic stroke
Phromminitikul et al. 2011 (24)	Thailand	RCT	221 vaccinated/218 unvaccinated	Inpatient	>50 years old	Any stroke
Lin et al. 2014 (12)	Taiwan	Case–control	Cases: 179 vaccinated/341 unvaccinated	Inpatient	≥ 65 years old	Hemorrhagic and Ischemic stroke
			Controls: 1,055 vaccinated/1,545 unvaccinated			
Siriwardena et al. 2014 (15)	UK	Case–control	Cases: 13,547 vaccinated/13,201unvaccinated	Outpatient	≥18 years old	Any stroke
			Controls: 13,605 vaccinated/13,143 unvaccinated			
Lavallee et al. 2014 (18)	International	Pooled analysis of two prospective cohorts and one RCT	5,054 vaccinated/5,054 unvaccinated	Multicenter	≥18 years old	Ischemic stroke
Vamos et al. 2016 (25)	UK	Retrospective cohort	114,198 vaccinated/59,882 unvaccinated	Patients with type 2 diabetes	≥18 years old	Any stroke
Liu et al. 2017 (26)	Taiwan	Retrospective	2,547 vaccinated/4,023 unvaccinated	Afib patients	≥55 years old	Hemorrhagic stroke
Kao et al. 2017 (27)	Taiwan	Retrospective	2,547 vaccinated/4,023 unvaccinated	Afib patients	≥55 years old	Ischemic stroke
Chiang et al. 2017 (28)	Taiwan	Case–control	Cases: 29,046 vaccinated/51,317 unvaccinated	Inpatient	≥65 years old	Ischemic stroke
			Controls: 33,285 vaccinated/47,078 unvaccinated			
Christiansen et al. 2019 (29)	Denmark	Retrospective cohort	30,877 vaccinated/30,877 unvaccinated	Inpatient	≥65 years old	any stroke
Lam et al. 2019 (30)	Taiwan	Retrospective cohort	25,248 vaccinated/25,248 unvaccinated	Inpatient	≥66 years old	Hemorrhagic and Ischemic stroke
Chang et al. 2020 (31)	Taiwan	Retrospective cohort	136,448 vaccinated/136,448 unvaccinated	Elderly patients with disability	≥65 years old	Hemorrhagic and Ischemic stroke
Fröbert et al. 2021 (13)	Sweden, Denmark, Norway, Latvia, UK, Czech Republic, Bangladesh, and Australia	RCT	1,272 vaccinated/1,260 unvaccinated	MI patients	≥18 years old	Any stroke
Chen et al. 2022 (32)	Taiwan	Retrospective cohort	2,551 vaccinated/2,551 unvaccinated	Female COPD patients	≥65 years old	Any stroke
Loeb et al. 2022 (33)	Asia (China, India, and Philippines), the Middle East (KSA and UAE), and Africa (Kenya, Mozambique, Nigeria, Uganda, and Zambia)	RCT	2,560 vaccinated/2,569 unvaccinated	HF patients	≥18 years old	Any stroke
Pang et al. 2022 (34)	China	Retrospective cohort	95,060 vaccinated/618,428 unvaccinated	Inpatient	≥60 years old	Hemorrhagic and Ischemic stroke
Holodinsky et al. 2022 (35)	Canada	Retrospective cohort	1,769,565 vaccinated/2,371,644 unvaccinated	Papulation based	≥18 years old	Hemorrhagic and Ischemic stroke
Rodríguez-Martín et al. 2022 (36)	Spain	Case–control	Cases: 5,930 vaccinated/8,392 unvaccinated	Outpatient	40–99 years old	Ischemic stroke
			Controls: 28,975 vaccinated/42,635 unvaccinated			

OR, odd ratio; RCT, randomized controlled trial; NR, not reported; UK, United Kingdom; USA, United States of America; KSA, Kingdom of Saudi Arabia; UAE, United Arab Emirates; HF, Heart failure; COPD, Chronic obstructive pulmonary disease; Afib, Atrial fibrillation; MI, Myocardial infarction.

### 3.3 Risk of bias

The risk of bias of the three RCTs was assessed by using the Revised Cochrane risk-of-bias tool for randomized trials, and two RCTs showed a low risk of bias across all the domains. However, Phromminitikul et al. had some concerns in the randomization domain (Supplementary Table S1). The risk of bias assessment for cohort and case–control studies was done using the Newcastle-Ottawa scale. For cohort studies, most showed a low risk of bias (14, 23, 25, 27, 29, 30, 32, 34) except for Liu et al. (26) Nichol et al. (20), Holodinsky et al. (35) and Chang et al. (31) showed a moderate risk of bias (Supplementary Table S2). For case–control studies, most showed a moderate risk of bias (15–17, 19, 22, 28), while (10, 12, 36) showed a low risk of bias, and Grau et al. (21) showed a high risk (Supplementary Table S3).

### 3.4 Association between influenza vaccine and incidence/hospitalization due to stroke

**Figure 2:**
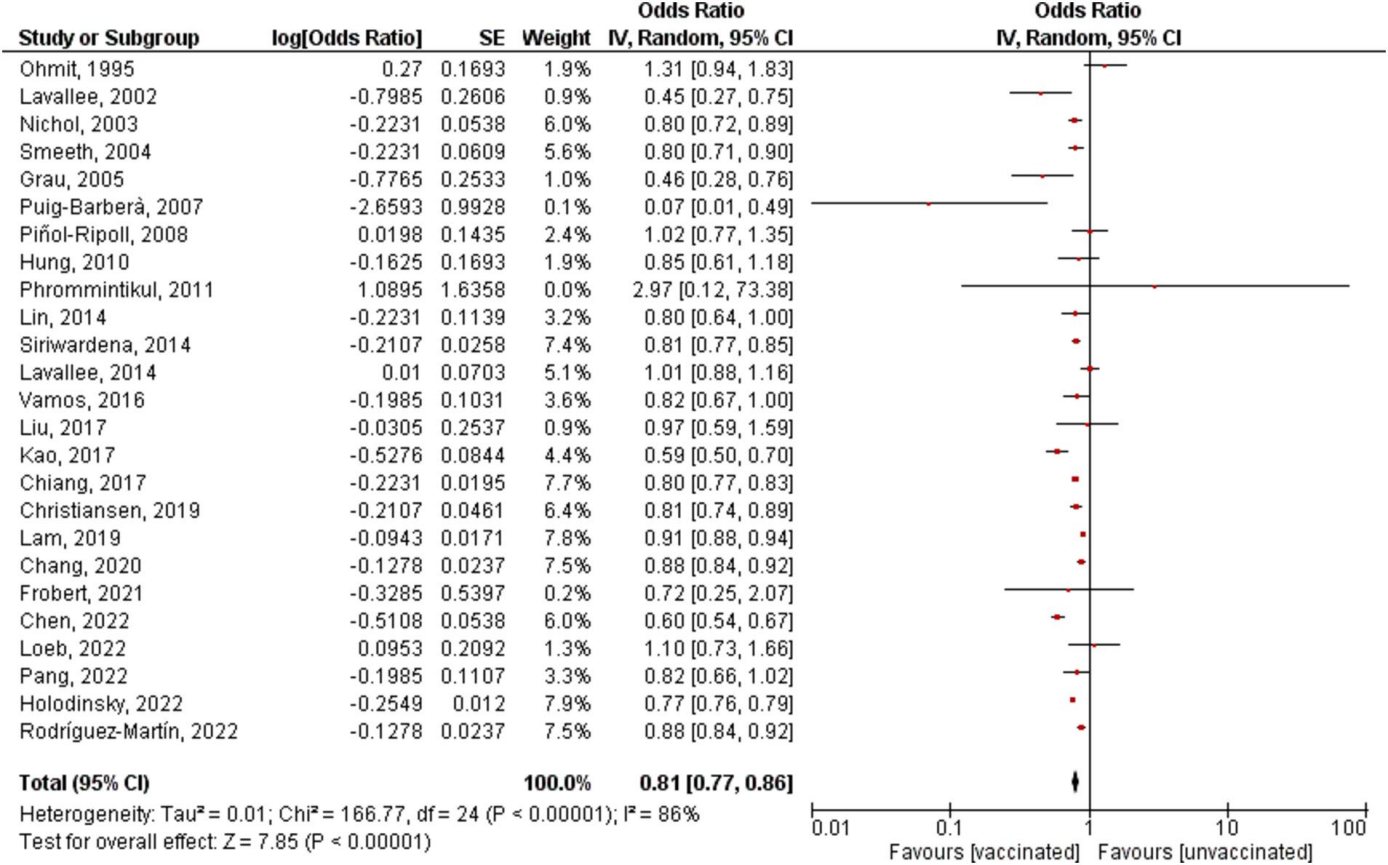
Forest plot showing the effectiveness of influenza vaccine on stroke incidence/hospitalization. CI, confidence interval.

**Figure 3:**
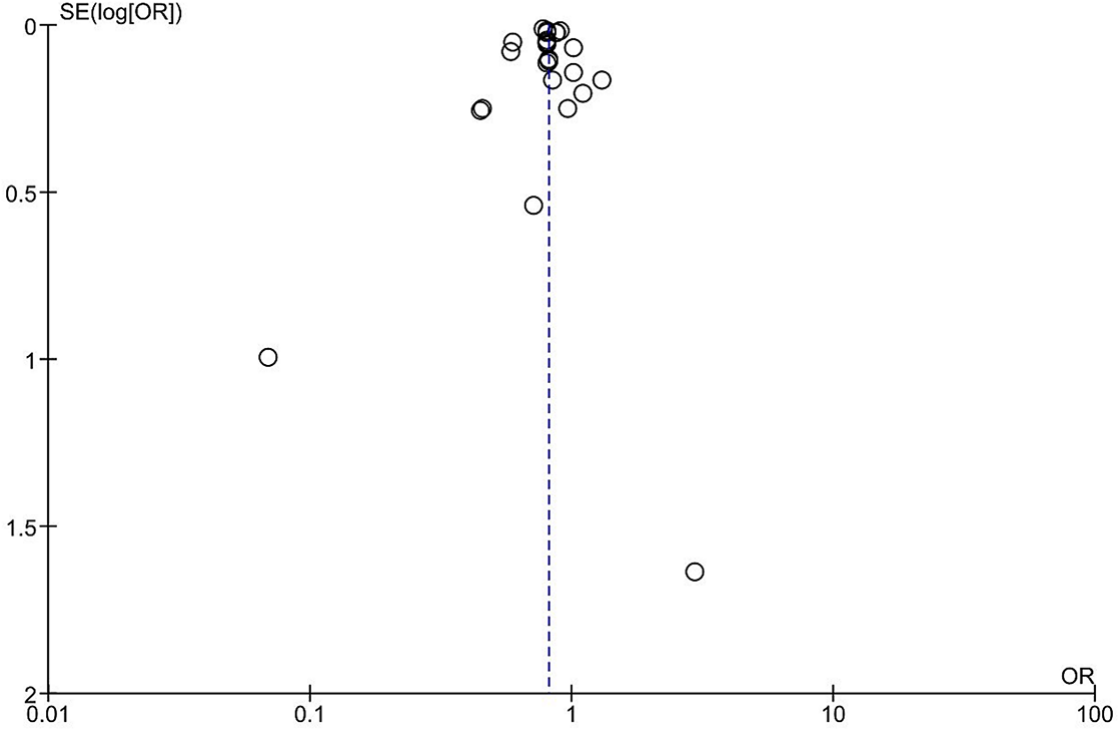
Funnel plot. OR, odds ratio.

All the studies showed an overall significant decrease in the risk of stroke incidence/hospitalization among vaccinated patients compared to unvaccinated patients; however, there is significant heterogeneity (OR = 0.81, 95% CI [0.77–0.86], *p* = 0.00001, I^2^ = 86%; Figure 2). Sensitivity analysis was conducted by removing each study at a time, the highest change of heterogeneity was noted by removing Fai Lam study with OR equal to 0.81 (95% CI [0.77–0.85], p = 0.00001, I^2^ = 81%); however, it was insignificant (Supplementary Figure S1). Therefore, the result should be interpreted with caution. The funnel plot revealed that there was no evidence of publication bias, and the studies included in the analysis were distributed in a symmetrical pattern (Figure 3).

### 3.5 Relationship between mortality in stroke patients and influenza vaccination

**Figure 4:**
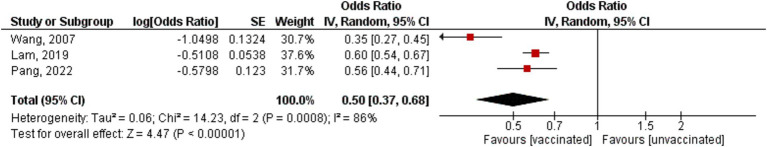
Forest plot showing the effectiveness of influenza vaccine on mortality in stroke patients. CI, confidence interval.

Only Three studies assessed the mortality incidence in stroke patients who received the influenza vaccine and showed that the vaccine decreases the occurrence of mortality among stroke patients (OR = 0.50, 95% CI [0.37–0.68], *p* = 0.00001, I^2^ = 86%; Figure 4). Additionally, sensitivity analysis was performed and by removing Wang’s study, the heterogeneity was 0% (OR = 0.59, 95% CI [0.54–0.65], *p* = 0.00001, I^2^ = 0%; Supplementary Figure S2).

### 3.6 Subgroup analysis

**Figure 5:**
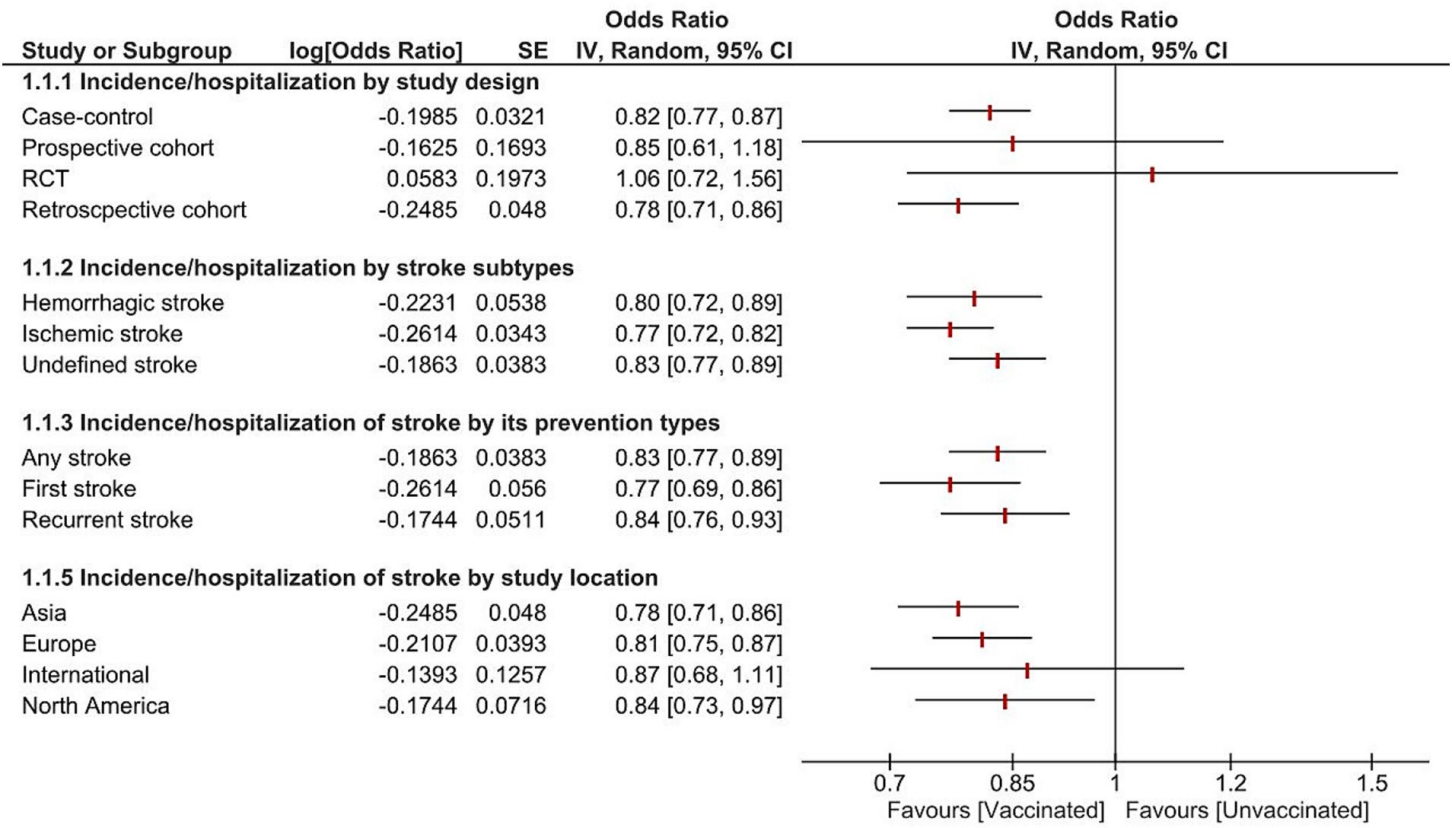
Forest plot showing the effectiveness of influenza vaccine on stroke incidence/hospitalization by study design, stroke subtypes, prevention, and location. CI, confidence interval.

Subgroup analysis was reported based on study design, stroke subtypes, prevention, and location (Figure 5). The pooled analysis showed significant reduction of stroke occurrence in vaccinated patients across stroke subtypes including ischemic stroke, hemorrhagic stroke, and undefined stroke. Additionally, all the studies exhibited a significant reduction in stroke incidence by subgroup analysis of first stroke, recurrent stroke, and any stroke. Furthermore, all the study designs revealed a reduction in the incidence of stroke, except for the RCTs and prospective studies; there was no significant difference (OR = 1.06, 95% CI [0.72–1.56], *p* = 0.78, I^2^ = 0%), and (OR = 0.85, 95% CI [0.61–1.18], *p* = 0.34), respectively. Depending on study location there was a significant reduction in stroke incidence in North America, Europe, and Asia. However, Studies conducted internationally did not reveal a statistically significant decrease in stroke incidence (OR = 0.87, 95% CI [0.68–1.11], *p* = 0.25, I^2^ = 86%).

### 3.7 Relationship between the risk of stroke and vaccination in patients with atrial fibrillation, diabetes, COPD, or hypertension

For atrial fibrillation patients, the pooled estimate of three studies showed that the incidence of stroke is decreased in those who received the vaccine (OR = 0.68, 95% CI [0.57–0.81], *p* = 0.0001, I^2^ = 65%; Supplementary Figure S3). For COPD patients, three studies showed a reduction in stroke occurrence in those who received the vaccine (OR = 0.70, 95% CI [0.61–0.81], *p* = 0.00001, I^2^ = 85%; Supplementary Figure S4). As for patients with diabetes, the pooled analysis of four studies also revealed a reduction in stroke occurrence in those who received the vaccine (OR = 0.76, 95% CI [0.66–0.87], p = 0.0001, I^2^ = 92%; Supplementary Figure S5). Moreover, another three studies showed that the vaccine reduced the occurrence of stroke in hypertensive patients (OR = 0.76, 95% CI [0.70–83], p = 0.00001, I^2^ = 93%; Supplementary Figure S6).

## 4 Discussion

The potential impact of influenza vaccination on stroke incidence, hospitalization, and mortality is the focus of our research. This systematic review and meta-analysis aimed to explore the potential benefits of the influenza vaccine beyond its established role in preventing influenza, by examining its effects on stroke prevention. To our knowledge, this meta-analysis included the largest number of studies to date and further supports conclusions made by previous meta-analyses (37, 38). Our findings suggest that receiving the influenza vaccine has a significant impact on lowering the incidence of stroke. Our research revealed a consistent reduction in stroke mortality among patients who received the influenza vaccine. Although this aspect was only studied in a limited number of studies, the results consistently pointed toward a protective effect of the vaccine on stroke mortality. Additionally, our review showed that the influenza vaccine may be particularly beneficial for patients with comorbidities such as atrial fibrillation, COPD, DM, and hypertension, as those who received the vaccine consistently showed a lower incidence of stroke. A study of particular significance is the study conducted by Holodinsky et al. (35) that showed the protective effect of the vaccine applied to the entire population of Alberta, Canada. The study included the largest sample size of over 4 million individuals, over a 9-year period from 2009 to 2018. The key finding from this comprehensive study is that influenza vaccination significantly reduced the risk of stroke across all types in the entire adult population of Alberta, Canada and the results were not influenced by the COVID-19, as the study was conducted prior to the pandemic. This aligns with the results of our meta-analysis, highlighting the substantial benefits of influenza vaccination in preventing stroke.

Although our study and recent studies indicate that the influenza vaccine may reduce stroke incidence, the exact process through which it does this is not fully understood. Nevertheless, several credible explanations have been suggested and studied. One leading explanation is that the vaccine decreases inflammation and endothelial dysfunction, both of which significantly contribute to atherosclerosis, the development and rupture of plaques which lead to strokes (39). Experimental studies in animal models with hypercholesterolemia have also shown a decrease in atherosclerosis following vaccination (40). A case crossover study on 5,888 patients at 4 different locations in the US further supports the explanation that systemic infections like influenza can provoke an immune response that heightens stroke risk, potentially through the instigation of a procoagulant state, especially during episodes of severe inflammation, organ damage, and sepsis (41). The influenza vaccine can potentially prevent these severe infections from occurring in the first place. However, the protective effect of influenza appears to be multifactorial. Our review includes studies such as the study by Rodriguez-Martin et al. (36) which found that the vaccine provides stroke protection even during pre-epidemic periods, which would not be explained by the vaccine’s protection against infection. This suggests that the vaccine might indirectly prevent stroke, as the authors propose; this may be due to unmeasured confounding factors. Although, the pneumococcal vaccine in the same study did not show a similar protective effect against stroke. If unmeasured confounding factors were the reason, both vaccines would be expected to demonstrate comparable associations with stroke prevention.

Some studies in our analysis showed no benefit from the influenza vaccine. One of which is a study by Loeb et al. (33) which did not demonstrate a significant impact of vaccination on stroke incidence. The vaccine’s protective effect was more pronounced when influenza activity was high but had a small effect during periods of low circulation of influenza. This lack of effect on stroke might be attributed to the fact that measurements during non-peak periods of influenza circulation affected the result. However, the study still showed effectiveness of the vaccine in reducing pneumonia and heart failure hospitalizations during peak influenza seasons. Piñol-Ripoll et al. (17) also found no significant stroke prevention benefit from the influenza vaccination. The authors suggested this might be either due to the vaccine’s ineffectiveness in preventing ischemic stroke, a small potential benefit from the vaccine, or inefficiency in preventing acute infections in the study’s specific patient group as a much higher percentage of vaccinated patients experienced acute infections compared to the unvaccinated group. Lavalle et al. (18) is another study where the influenza vaccine has not significantly reduced stroke incidence. Their study, predominantly derived from observational analyses, included a population where the majority was on antithrombotic therapies, antihypertensive therapies, and lipid lowering drugs with regular follow-up visits in specialized centers. This may have left little room for any additional benefit from the influenza vaccination, potentially limiting its efficacy in preventing strokes among this group. Furthermore, the absence of information on the matching between circulating virus strains and vaccine antigens in different countries studied may have impacted the results. Another study by Ohmit et al. (19) explored no benefit of influenza vaccination in decreasing the risk of stroke occurrence. This could be explained by the fact that Ohmit et al. included patients with cancer which affects the immune system markedly. Therefore, the body loses its ability to produce antibodies against influenza vaccine components which could make the vaccine less effective. Additionally, Phromminitikul et al. (24) could not investigate the effect of the relationship between the vaccine and stroke incidence since the event was very rare to extract a proper conclusion. The varying results underscore the complexity of the relationship between the influenza vaccine and stroke incidence, highlighting that there are likely multiple factors that might affect the effectiveness of the influenza vaccine in preventing stroke.

Our research offers valuable insights into the potential benefits of influenza vaccination in stroke prevention, however, there are several limitations that need to be considered. The significant heterogeneity identified in our study suggests differences in baseline patient characteristics, recall bias, and study designs. There were large differences between the types of patients and settings in the studies analyzed as shown in Table 1. Such variation between patient populations and characteristics is likely one of the sources for the significant heterogeneity. One source of bias that is discussed by Jackson et al. (42) in their paper is bias arising from the better overall health status of vaccinated individuals compared to unvaccinated ones. This bias likely inflates the perceived effectiveness of the vaccine, as evidenced by the lower risk of death and hospitalization in vaccinated seniors even in pre-epidemic periods. Moreover, Jackson et al. note that their attempts to adjust for this health status bias, did not adequately control for it, as reduction in death and hospitalizations remained significant in the pre-epidemic period when they would be expected to be insignificant. The failure of this adjustment method to account for bias might be because the diagnosis codes used do not effectively measure frailty or the intensity of illnesses. This indicates a need for more effective adjustment methods in future studies. Additionally, healthcare seeking behavior might also play a role in this observed bias. Vaccinated individuals might be more proactive in seeking medical care compared to unvaccinated individuals, which could further inflate the perceived effectiveness of the vaccine. Furthermore, most of the studies included in our analysis were observational, which may be subjected to various biases and confounding factors. In case–control studies conducted at an inpatient setting, where patients are selected retrospectively after the influenza season, the survival of patients at time of inclusion could lead to selection bias. Another limitation is the absence of studies from Australia, South America, or Africa, which may limit the generalizability of our results to these regions. Finally, it is essential to note that the pathogenicity and infectivity of the influenza virus can change from year to year, and the vaccine’s effectiveness may vary accordingly. Thus, our findings may not be applicable to all influenza seasons or strains. The limitations discussed make it difficult to establish a causal relationship between influenza vaccination and stroke risk. More randomized controlled trials are needed to control for patient characteristics such as frailty that are difficult to control for in other types of studies. In addition, future studies should explore the risk of stroke following influenza vaccination in the pre-epidemic, epidemic, and post-epidemic periods.

Current clinical guidelines recommend the influenza vaccine for patients with cardiovascular disease (43); however, no such recommendation is made for patients with cerebrovascular disease. Considering the results of our study and the current body of evidence that points toward a clear protective effect of the influenza vaccine against stroke, we believe that for all patients without apparent contraindication to the vaccine, who are at risk of stroke, or stroke survivors, should be encouraged to receive the influenza vaccine to reduce mortality, hospitalization, and morbidity. Future guidelines for the treatment and management of stroke patients or at-risk patients should strongly consider recommending the influenza vaccine, especially for patients with atrial fibrillation, COPD, DM, and hypertension. Implementing this recommendation, particularly for patients with risk factors for stroke, may have a significant effect on public health, potentially leading to a decrease in stroke incidence, along with a possible reduction in hospital admissions. It may also lessen the health complications related to stroke and lower the need for extensive rehabilitation programs. This could potentially ease the considerable financial and personal strain placed on healthcare systems, patients, and their families.

## 5 Conclusion

Influenza vaccination has a significant impact on mitigating both the incidence and mortality of stroke, particularly among patients with risk factors for stroke. The current clinical guidelines should be expanded to encourage influenza vaccination for stroke survivors and patients at risk for stroke. Further randomized controlled trials are needed to confirm the link between influenza vaccination and stroke risk reduction. Additional studies should focus on understanding the precise mechanisms involved in the protective effect of the influenza vaccine against stroke.

## Data availability statement

The raw data supporting the conclusions of this article will be made available by the authors, without undue reservation.

## Author contributions

JZ: Data curation, Investigation, Methodology, Software, Supervision, Writing – original draft, Writing – review & editing, Conceptualization, Project administration. HS: Conceptualization, Formal Analysis, Investigation, Methodology, Software, Supervision, Writing – original draft, Writing – review & editing. MA: Data curation, Investigation, Writing – original draft. DF: Data curation, Investigation, Writing – original draft. LA: Data curation, Investigation, Writing – original draft. TB: Data curation, Investigation, Writing – original draft. NA: Data curation, Investigation, Writing – original draft. MB: Data curation, Investigation, Writing – original draft. SA: Conceptualization, Project administration, Supervision, Validation, Writing – review & editing. SM: Conceptualization, Project administration, Supervision, Validation, Writing – review & editing.

## References

[1] Murphy SJX Werring DJ. Stroke: causes and clinical features. Medicine. (2020) 48:561–6. doi: 10.1016/j.mpmed.2020.06.002, PMID: 32837228 PMC7409792

[2] Feigin VL Stark BA Johnson CO Roth GA Bisignano C Abady GG . Global, regional, and national burden of stroke and its risk factors, 1990–2019: a systematic analysis for the global burden of disease study 2019. Lancet Neurol. (2021) 20:795–820. doi: 10.1016/S1474-4422(21)00252-0, PMID: 34487721 PMC8443449

[3] Donkor ES. Stroke in the 21st century: A snapshot of the burden, epidemiology, and quality of life. Stroke Res Treat. (2018) 2018:1–10. doi: 10.1155/2018/3238165, PMID: 30598741 PMC6288566

[4] Alqahtani BA Alenazi AM Hoover JC Alshehri MM Alghamdi MS Osailan AM . Incidence of stroke among Saudi population: a systematic review and meta-analysis. Neurol Sci. (2020) 41:3099–104. doi: 10.1007/s10072-020-04520-4, PMID: 32564272

[5] Tavabe NR Kheiri S Dehghani M Mohammadian-Hafshejani A. A systematic review and Meta-analysis of the relationship between receiving the flu vaccine with acute cerebrovascular accident and its hospitalization in the elderly. Biomed Res Int. (2023) 2023:1–11. doi: 10.1155/2023/2606854, PMID: 36814798 PMC9940958

[6] Boehme AK Luna J Kulick ER Kamel H Elkind MSV. Influenza-like illness as a trigger for ischemic stroke. Ann Clin Transl Neurol. (2018) 5:456–63. doi: 10.1002/acn3.545, PMID: 29687022 PMC5899905

[7] Nguyen JL Yang W Ito K Matte TD Shaman J Kinney PL. Seasonal influenza infections and cardiovascular disease mortality. JAMA Cardiol. (2016) 1:274–81. doi: 10.1001/jamacardio.2016.0433, PMID: 27438105 PMC5158013

[8] McColl BW Allan SM Denes A Lawrence CB. Letter by McColl et al regarding article, “influenza virus infection aggravates stroke outcome.”. Stroke. (2011) 42:1276. doi: 10.1161/STROKEAHA.111.621276 21719776

[9] Naghavi M Wyde P Litovsky S Madjid M Akhtar A Naguib S . Influenza infection exerts prominent inflammatory and thrombotic effects on the atherosclerotic plaques of apolipoprotein E–deficient mice. Circulation. (2003) 107:762–8. doi: 10.1161/01.CIR.0000048190.68071.2B 12578882

[10] Smeeth L Thomas SL Hall AJ Hubbard R Farrington P Vallance P. Risk of myocardial infarction and stroke after acute infection or vaccination. N Engl J Med. (2004) 351:2611–8. doi: 10.1056/NEJMoa041747, PMID: 15602021

[11] Barnes M Heywood AE Mahimbo A Rahman B Newall AT Macintyre CR. Acute myocardial infarction and influenza: a meta-analysis of case–control studies. Heart. (2015) 101:1738–47. doi: 10.1136/heartjnl-2015-307691, PMID: 26310262 PMC4680124

[12] Lin H-C Chiu H-F Ho S-C Yang C-Y. Association of Influenza Vaccination and Reduced Risk of stroke hospitalization among the elderly: A population-based case-control study. Int J Environ Res Public Health. (2014) 11:3639–49. doi: 10.3390/ijerph110403639, PMID: 24699027 PMC4025018

[13] Fröbert O Götberg M Erlinge D Akhtar Z Christiansen EH MacIntyre CR . Influenza vaccination after myocardial infarction: A randomized, double-blind, placebo-controlled, multicenter trial. Circulation. (2021) 144:1476–84. doi: 10.1161/CIRCULATIONAHA.121.057042, PMID: 34459211

[14] Wang C-S Wang S-T Lai C-T Lin L-J Chou P. Impact of influenza vaccination on major cause-specific mortality. Vaccine. (2007) 25:1196–203. doi: 10.1016/j.vaccine.2006.10.015, PMID: 17097773

[15] Siriwardena AN Asghar Z Coupland CCA. Influenza and pneumococcal vaccination and risk of stroke or transient ischaemic attack—matched case control study. Vaccine. (2014) 32:1354–61. doi: 10.1016/j.vaccine.2014.01.029, PMID: 24486370

[16] Lavallée P Perchaud V Gautier-Bertrand M Grabli D Amarenco P. Association between influenza vaccination and reduced risk of brain infarction. Stroke. (2002) 33:513–8. doi: 10.1161/hs0202.102328, PMID: 11823662

[17] Piñol-Ripoll G de la Puerta I Santos S Purroy F Mostacero E. Chronic bronchitis and acute infections as new risk factors for ischemic stroke and the lack of protection offered by the influenza vaccination. Cerebrovasc Dis. (2008) 26:339–47. doi: 10.1159/000151636 18728360

[18] Lavallee PC Labreuche J Fox KM Lavados P Mattle H Steg PG . Influenza vaccination and cardiovascular risk in patients with recent TIA and stroke. Neurology. (2014) 82:1905–13. doi: 10.1212/WNL.0000000000000456, PMID: 24789867

[19] Ohmit SE Furumoto-Dawson A Monto AS Fasano N. Influenza vaccine use among an el-derly population in a community in-tervention. Am J Prev Med. (1995) 11:271–6. doi: 10.1016/S0749-3797(18)30457-4 7495605

[20] Nichol KL Nordin J Mullooly J Lask R Fillbrandt K Iwane M. Influenza vaccination and reduction in hospitalizations for cardiac disease and stroke among the elderly. N Engl J Med. (2003) 348:1322–32. doi: 10.1056/NEJMoa025028, PMID: 12672859

[21] Grau AJ Fischer B Barth C Ling P Lichy C Buggle F. Influenza vaccination is associated with a reduced risk of stroke. Stroke. (2005) 36:1501–6. doi: 10.1161/01.STR.0000170674.45136.80 15947266

[22] Puig-Barberà J Díez-Domingo J Varea ÁB Chavarri GS Rodrigo JAL Hoyos SP . Effectiveness of MF59™-adjuvanted subunit influenza vaccine in preventing hospitalisations for cardiovascular disease, cerebrovascular disease and pneumonia in the elderly. Vaccine. (2007) 25:7313–21. doi: 10.1016/j.vaccine.2007.08.039, PMID: 17889411

[23] Hung IFN Leung AYM Chu DWS Leung D Cheung T Chan C . Prevention of acute myocardial infarction and stroke among elderly persons by dual pneumococcal and influenza vaccination: A prospective cohort study. Clin Infect Dis. (2010) 51:1007–16. doi: 10.1086/656587, PMID: 20887208

[24] Phrommintikul A Kuanprasert S Wongcharoen W Kanjanavanit R Chaiwarith R Sukonthasarn A. Influenza vaccination reduces cardiovascular events in patients with acute coronary syndrome. Eur Heart J. (2011) 32:1730–5. doi: 10.1093/eurheartj/ehr004, PMID: 21289042

[25] Vamos EP Pape UJ Curcin V Harris MJ Valabhji J Majeed A . Effectiveness of the influenza vaccine in preventing admission to hospital and death in people with type 2 diabetes. Can Med Assoc J. (2016) 188:E342–51. doi: 10.1503/cmaj.151059, PMID: 27455981 PMC5047834

[26] Liu J-C Wang T-J Sung L-C Kao P-F Yang T-Y Hao W-R . Influenza vaccination reduces hemorrhagic stroke risk in patients with atrial fibrillation: A population-based cohort study. Int J Cardiol. (2017) 232:315–23. doi: 10.1016/j.ijcard.2016.12.074, PMID: 28089151

[27] Kao P-F Liu J-C Hsu Y-P Sung L-C Yang T-Y Hao W-R . Influenza vaccination might reduce the risk of ischemic stroke in patients with atrial fibrillation: A population-based cohort study. Oncotarget. (2017) 8:112697–711. doi: 10.18632/oncotarget.22352 29348857 PMC5762542

[28] Chiang M-H Wu H-H Shih C-J Chen Y-T Kuo S-C Chen T-L. Association between influenza vaccination and reduced risks of major adverse cardiovascular events in elderly patients. Am Heart J. (2017) 193:1–7. doi: 10.1016/j.ahj.2017.07.020, PMID: 29129247

[29] Christiansen CF Thomsen RW Schmidt M Pedersen L Sørensen HT. Influenza vaccination and 1-year risk of myocardial infarction, stroke, heart failure, pneumonia, and mortality among intensive care unit survivors aged 65 years or older: a nationwide population-based cohort study. Intensive Care Med. (2019) 45:957–67. doi: 10.1007/s00134-019-05648-4, PMID: 31187170

[30] Lam F Chen T-L Shih C-C Lin C-S Yeh C-C Lee Y-J . Protective effect of influenza vaccination on outcomes in geriatric stroke patients: A nationwide matched cohort study. Atherosclerosis. (2019) 282:85–90. doi: 10.1016/j.atherosclerosis.2019.01.008, PMID: 30711633

[31] Chang Y-C Yu-Tung H Chen L-S Tung H-J Huang K-H Ernawaty E . Protective effect of seasonal influenza vaccination in elderly individuals with disability in Taiwan: A propensity score-matched, Nationwide, population-based cohort study. Vaccines. (2020) 8:140. doi: 10.3390/vaccines8010140, PMID: 32235779 PMC7157623

[32] Chen CC Lin CH Chiu CC Yang TY Hsu MH Wang YH . Influenza vaccination and risk of stroke in women with chronic obstructive pulmonary disease: A Nationwide, population-based, propensity-matched cohort study. Front Med. (2022) 9:811021. doi: 10.3389/fmed.2022.811021 PMC9160371 35665329

[33] Loeb M Roy A Dokainish H Dans A Palileo-Villanueva LM Karaye K . Influenza vaccine to reduce adverse vascular events in patients with heart failure: a multinational randomised, double-blind, placebo-controlled trial. Lancet Glob Health. (2022) 10:e1835–44. doi: 10.1016/S2214-109X(22)00432-6, PMID: 36400089

[34] Pang Y Liu X Liu G Lv M Lu M Wu J . Effectiveness of influenza vaccination on in-hospital death and recurrent hospitalization in older adults with cardiovascular diseases. Int J Infect Dis. (2022) 122:162–8. doi: 10.1016/j.ijid.2022.05.059, PMID: 35654282

[35] Holodinsky JK Zerna C Malo S Svenson LW Hill MD. Association between influenza vaccination and risk of stroke in Alberta, Canada: a population-based study. Lancet Public Health. (2022) 7:e914–22. doi: 10.1016/S2468-2667(22)00222-5 36334607

[36] Rodríguez-Martín S Barreira-Hernández D Gil M García-Lledó A Izquierdo-Esteban L De Abajo F. Influenza vaccination and risk of ischemic stroke. Neurology. (2022) 99:e2149–60. doi: 10.1212/WNL.0000000000201123, PMID: 36240087

[37] Lee KR Bae JH Hwang IC Kim KK Suh HS Ko KD. Effect of influenza vaccination on risk of stroke: A systematic review and Meta-analysis. Neuroepidemiology. (2017) 48:103–10. doi: 10.1159/000478017, PMID: 28628919

[38] Tsivgoulis G Katsanos AH Zand R Ishfaq MF Malik MT Karapanayiotides T . The association of adult vaccination with the risk of cerebrovascular ischemia: A systematic review and meta-analysis. J Neurol Sci. (2018) 386:12–8. doi: 10.1016/j.jns.2018.01.007, PMID: 29406959

[39] Libby P Theroux P. Pathophysiology of coronary artery disease. Circulation. (2005) 111:3481–8. doi: 10.1161/CIRCULATIONAHA.105.537878 15983262

[40] Hansson GK Nilsson J. Vaccination against atherosclerosis? Induction of atheroprotective immunity. Semin Immunopathol. (2009) 31:95–101. doi: 10.1007/s00281-009-0151-x, PMID: 19468734

[41] Elkind MSV Carty CL O’Meara ES Lumley T Lefkowitz D Kronmal RA . Hospitalization for infection and risk of acute ischemic stroke. Stroke. (2011) 42:1851–6. doi: 10.1161/STROKEAHA.110.608588 21546476 PMC3125478

[42] Jackson LA Jackson ML Nelson JC Neuzil KM Weiss NS. Evidence of bias in estimates of influenza vaccine effectiveness in seniors. Int J Epidemiol. (2006) 35:337–44. doi: 10.1093/ije/dyi274, PMID: 16368725

[43] Grohskopf LA Blanton LH Ferdinands JM Chung JR Broder KR Talbot HK . Prevention and Control of Seasonal Influenza with Vaccines: Recommendations of the Advisory Committee on Immunization Practices — United States, 2022–23 Influenza Season. MMWR Recomm Rep. (2022) 71:1–28. doi: 10.15585/mmwr.rr7101a1 PMC9429824 36006864

