# Supplementary material for: Influenza vaccine effect on risk of stroke occurrence: a systematic review and meta-analysis
Source: Front Neurol. 2024 Jan 10;14:1324677. doi: 10.3389/fneur.2023.1324677 (PMC10806129; doi:10.3389/fneur.2023.1324677)
Supplement: Supplementary file 1 [file Data_Sheet_1.docx]

**Supplementary materials**

**Supplementary figure** **S1.** Sensitivity analysis by removing Lam 2019 for incidence or hospitalization of stroke.


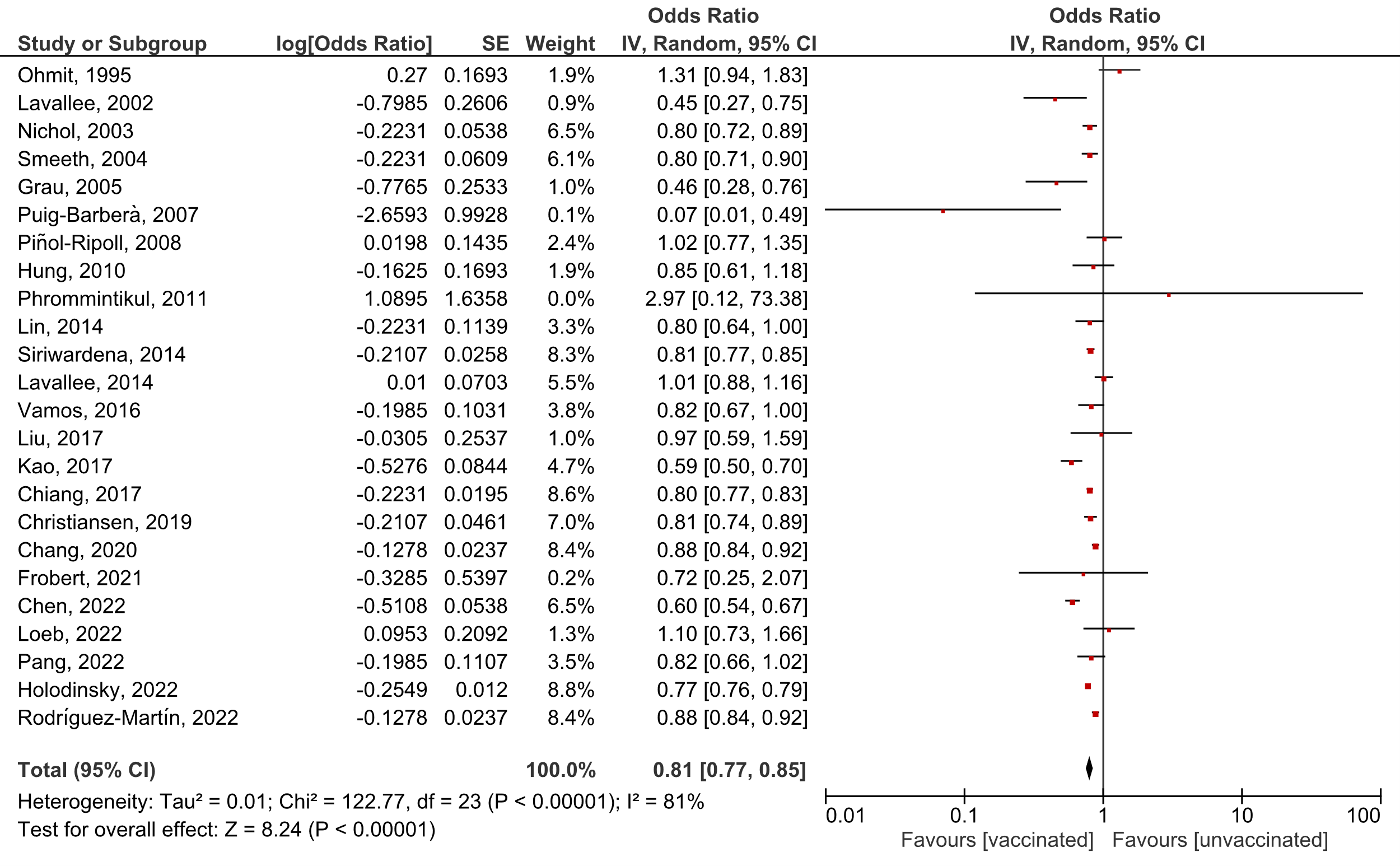


**Supplementary figure** **S2.** Sensitivity analysis of mortality by removing Wang 2007.


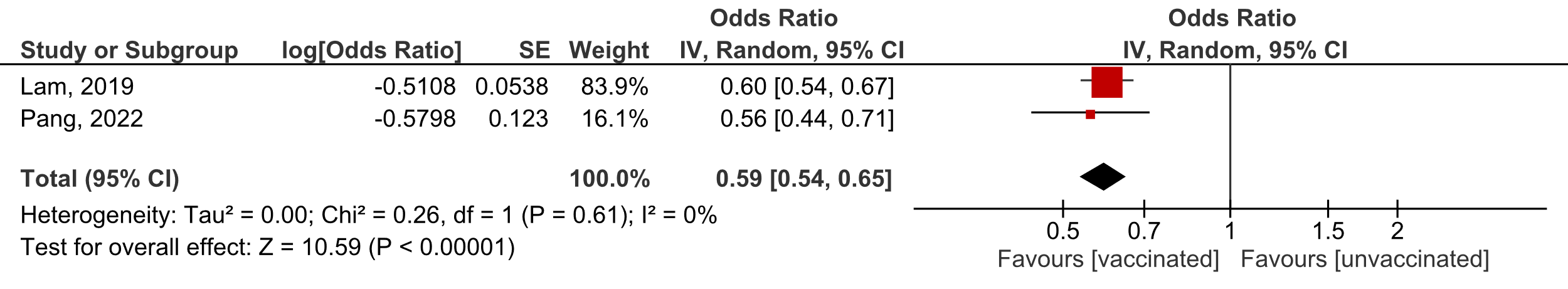


**Supplementary figure S3.** Risk of stroke in atrial fibrillation patients.


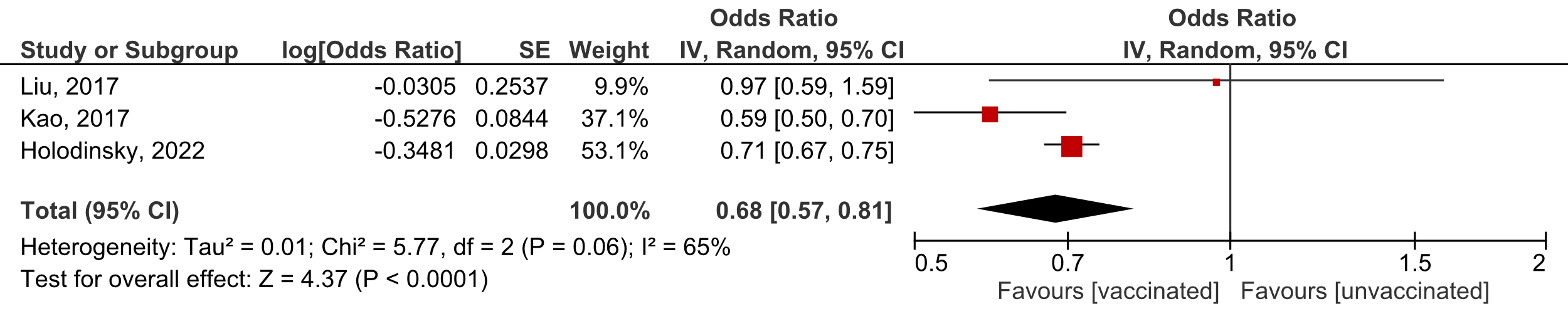


**Supplementary figure S4.** Risk of stroke in COPD patients.


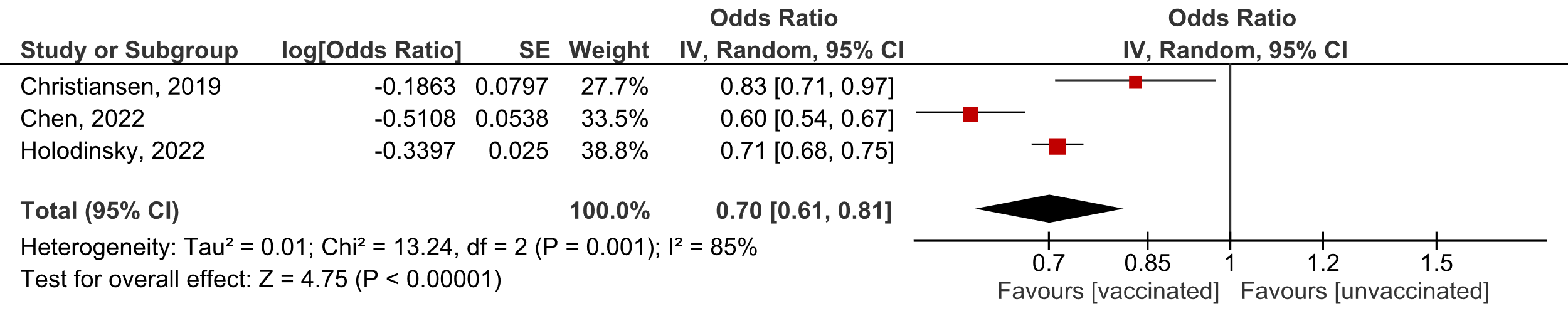


**Supplementary figure S5.** Risk of stroke in DM patients.


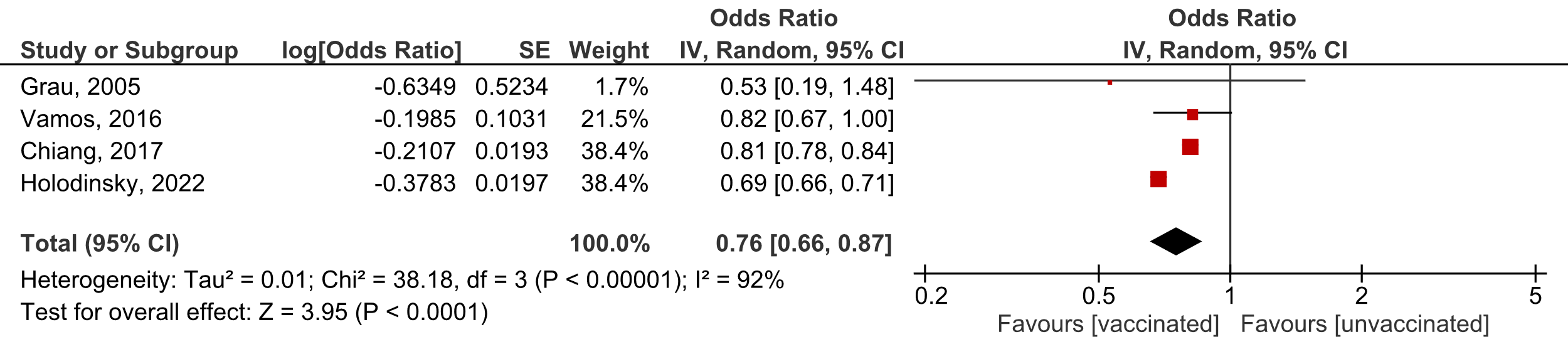


**Supplementary figure S6.** Risk of stroke in HTN patients.


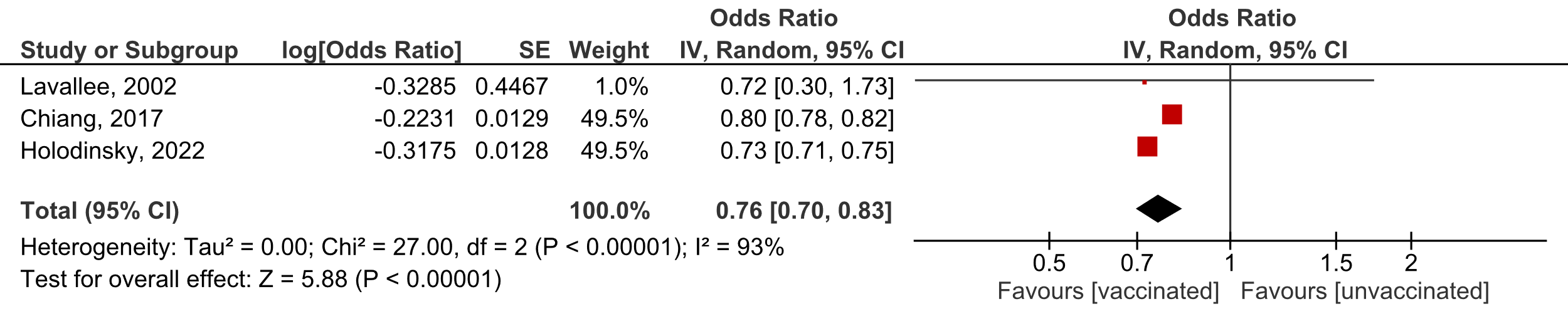


**Table. S1** Risk of bias assessment for randomized controlled trials.


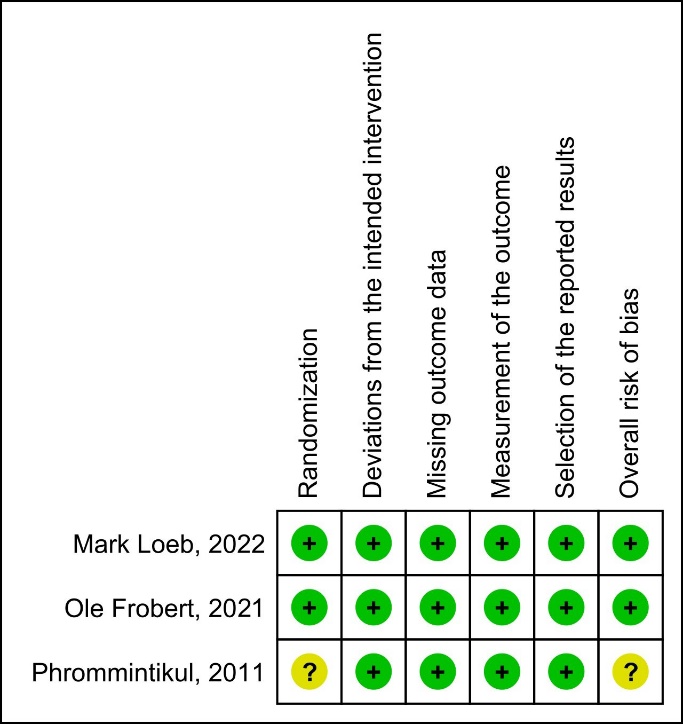


**Table. S2** Risk of bias assessment for cohort studies.

| **Study** | **Selection**  **(0-4)** | **Comparability**  **(0-2)** | **Outcome**  **(0-3)** | **Total score** | **Risk of bias** |
| --- | --- | --- | --- | --- | --- |
| Nichol et al. | 2 | 0 | 2 | 4 | Moderate |
| Wang et al. | 3 | 1 | 3 | 7 | Low |
| Hung et al. | 3 | 2 | 2 | 7 | Low |
| Vamos et al. | 3 | 1 | 3 | 7 | Low |
| Liu et al. | 3 | 0 | 3 | 6 | Moderate |
| Kao et al. | 3 | 2 | 2 | 7 | Low |
| Christiansen et al. | 3 | 2 | 2 | 7 | Low |
| Lam et al. | 3 | 2 | 3 | 8 | Low |
| Chang et al. | 2 | 1 | 2 | 5 | Moderate |
| Chen et al. | 3 | 2 | 2 | 7 | Low |
| Pang et al. | 3 | 1 | 3 | 7 | Low |
| Holodinsky et al. | 3 | 0 | 2 | 5 | Moderate |
| Mean | 2.83 | 1.17 | 2.42 | 77 |  |

Risk of bias (0-3: High, 4-6: Moderate, 7-9: Low

**Table. S3** Risk of bias assessment for case-control studies.

| **Study** | **Selection**  **(0-4)** | **Comparability**  **(0-2)** | **Exposure**  **(0-3)** | **Total score** | **Risk of bias** |
| --- | --- | --- | --- | --- | --- |
| Ohmit et al. | 2 | 1 | 1 | 4 | Moderate |
| Lavallee et al. | 2 | 1 | 1 | 4 | Moderate |
| Smeeth et al. | 3 | 1 | 3 | 7 | Low |
| Grau et al. | 2 | 0 | 1 | 3 | High |
| Puig-Barbera et al. | 2 | 0 | 2 | 4 | Moderate |
| Piñol-Ripoll et al. | 3 | 0 | 2 | 5 | Moderate |
| Lin et al. | 3 | 2 | 3 | 8 | Low |
| Siriwardena et al. | 2 | 1 | 3 | 6 | Moderate |
| Chiang et al.  Rodríguez-Martín | 2  3 | 2  2 | 2  2 | 6  7 | Moderate  Low |
| Mean | 2.3 | 0.89 | 2 | 47 |  |

Risk of bias (0-3: High, 4-6: Moderate, 7-9: Low)

**Table. 2** Risk of bias assessment for cohort and case-control studies.
